# Clinical Evaluation of Respiratory Rate Measurements on COPD (Male) Patients Using Wearable Inkjet-Printed Sensor

**DOI:** 10.3390/s21020468

**Published:** 2021-01-11

**Authors:** Ala’aldeen Al-Halhouli, Loiy Al-Ghussain, Osama Khallouf, Alexander Rabadi, Jafar Alawadi, Haipeng Liu, Khaled Al Oweidat, Fei Chen, Dingchang Zheng

**Affiliations:** 1 Mechatronics Engineering Department/NanoLab, School of Applied Technical Sciences, German Jordanian University, P.O. Box 35247, Amman 11180, Jordan; loiy.al-ghussain@uky.edu (L.A.-G.); Osama.Khallouf@gju.edu.jo (O.K.); jafar.alawadi@gmail.com (J.A.); 2 Institute of Microtechnology, Technische Universität Braunschweig, 38124 Braunschweig, Germany; 3 Faculty of Engineering, Middle East University, Amman 11831, Jordan; 4 Mechanical Engineering Department, University of Kentucky, Lexington, KY 40506, USA; 5 Department of Internal Medicine, Faculty of Medicine, University of Jordan, Amman 11942, Jordan; aly0152547@med.ju.edu.jo (A.R.); k.oweidat@ju.edu.jo (K.A.O.); 6 Research Centre for Intelligent Healthcare, Faculty of Health and Life Sciences, Coventry University, Coventry CV1 5FB, UK; haipeng.liu@coventry.ac.uk (H.L.); dingchang.zheng@coventry.ac.uk (D.Z.); 7 Department of Electrical and Electronic Engineering, Southern University of Science and Technology, Shenzhen 518055, China; fchen@sustech.edu.cn

**Keywords:** flexible and wearable sensors, respiratory rate, COPD patients, inkjet printing, clinical evaluation

## Abstract

Introduction: Chronic Obstructive Pulmonary Disease (COPD) is a progressive disease that causes long-term breathing problems. The reliable monitoring of respiratory rate (RR) is very important for the treatment and management of COPD. Based on inkjet printing technology, we have developed a stretchable and wearable sensor that can accurately measure RR on normal subjects. Currently, there is a lack of comprehensive evaluation of stretchable sensors in the monitoring of RR on COPD patients. We aimed to investigate the measurement accuracy of our sensor on COPD patients. Methodology: Thirty-five patients (Mean ± SD of age: 55.25 ± 13.76 years) in different stages of COPD were recruited. The measurement accuracy of our inkjet-printed (IJPT) sensor was evaluated at different body postures (i.e., standing, sitting at 90°, and lying at 45°) on COPD patients. The RR recorded by the IJPT sensor was compared with that recorded by the reference e-Health sensor using paired T-test and Wilcoxon signed-rank test. Analysis of variation (ANOVA) was performed to investigate if there was any significant effect of individual difference or posture on the measurement error. Statistical significance was defined as *p*-value less than 0.05. Results: There was no significant difference between the RR measurements collected by the IJPT sensor and the e-Health reference sensor overall and in three postures (*p* > 0.05 in paired T-tests and Wilcoxon signed-rank tests). The sitting posture had the least measurement error of −0.0542 ± 1.451 bpm. There was no significant effect of posture or individual difference on the measurement error or relative measurement error (*p* > 0.05 in ANOVA). Conclusion: The IJPT sensor can accurately measure the RR of COPD patients at different body postures, which provides the possibility for reliable monitoring of RR on COPD patients.

## 1. Introduction

Respiratory rate (RR) is a vital sign that is related to, therefore regulated by, multiple physiological and neural activities [1,2,3]. RR plays an important role in the detection of various cardiovascular and respiratory diseases, as well as relevant clinical events [4,5]. The variation of RR reflects the deterioration of respiratory diseases including asthma, chronic obstructive pulmonary disease (COPD), and other clinical conditions including fever, infection, and drug overdose [6]_._ Despite the development of RR measurement technologies, RR is still often neglected or inaccurately estimated in clinical practice [7].

Recently, wearable and flexible sensors (WFS) have been widely applied in medical and healthcare areas [8,9,10,11,12,13]. Especially, WFS can be used in the non-invasive monitoring of vital signs such as blood pressure [14], cardiac [15], and respiratory activities [15,16,17]. More specifically, several studies have investigated the use of WFS extensively in RR monitoring [18,19,20,21,22,23,24]. For instance, Jeong et al. [25] developed a fabric piezo-resistive sensor of RR and tested it in different physiological conditions, including resting, walking at two different speeds, and running activities. It was found that the averaged relative error of the sensor in RR measurement was about 3%. Lei et al. [26] proposed a PVDF-based (Piezoelectric-film) sensor patch and tested it in static (e.g., sitting or sleeping) and dynamic (e.g., walking) conditions. No significant measurement error was found when compared to the reference sensor (*p* > 0.05). Moreover, WFS can be used in the monitoring of vital signs on patients who have special medical conditions such as Chronic Obstructive Pulmonary Disease (COPD) [27].

COPD is a term to describe lung diseases such as chronic bronchitis, emphysema, and refractory asthma, and it is usually characterized by increasing breathlessness. The symptoms of COPD may differ between individuals, but commonly include shortness of breath, wheezing, and tightness in the chest [28]. The link between dyspnea and respiratory rate is of particular interest in COPD patients [3]. Moreover, it was suggested that hypnosis could contribute to the improvement of anxiety levels and breathing mechanics in severe COPD patients [29]. Several studies in the literature highlighted the importance of reliable RR monitoring in the early detection of COPD [30,31,32], where a RR over 25 breaths per min (bpm) was considered as a sign of COPD exacerbation [30,33]. Hence, the comprehensive evaluation of the measurement accuracy and the suitability of WFS in RR monitoring on COPD patients is important for clinical application.

Regarding RR measurement, the clinical evaluation of WFS was mainly performed on healthy subjects [34,35,36,37,38,39]. Few studies have investigated the accuracy of WFS in monitoring COPD patients’ vital signs [31,33,40]. Bellos et al. [31] investigated the accuracy of a wearable vest in monitoring different vital signs including RR on COPD patients. The system consisted of a wearable platform and some external devices connected to a smart device and a home patient monitor. The wearable platform along with the external devices extracted some useful information about the patient’s activities, living environment, and lifestyle. The collected data were then processed to evaluate the patient’s health status. The system achieved about 94% accuracy in RR monitoring. Moreover, Rubio et al. [33] evaluated the accuracy of five home-based RR measurement devices on 21 stable COPD patients during daily activities. The authors also investigated the acceptability (comfort) of the patients regarding wearing of the sensors. They concluded that the chest-band sensor was the most acceptable sensor for patients with good measurement accuracy with a bias of −1.60 bpm.

Al-Halhouli et al. [16] have presented the development of stretchable and wearable strain gauge sensor using inkjet printing technology for RR monitoring. The accuracy and performance of the developed sensor have been validated on healthy subjects at different body locations [41] and at different body postures [17]. They concluded that the inkjet-printed (IJPT) sensor was accurate and had a good potential for monitoring RR on non-healthy patients such as COPD patients. To the best of our knowledge, there is a lack of studies that investigated the accuracy of WFS for RR measurements on COPD patients at different postures. Hence, this study aimed to comprehensively evaluate the accuracy of the IJPT RR sensor- developed in [16] on COPD patients at different body postures at rest. Despite that, this study did not aim to investigate the ability of the developed sensor to diagnose COPD types, however the use of this sensor could help in reducing the frequency and severity of COPD exacerbation symptoms by early detection of abnormal physiological measurements including the respiratory rate [42] especially with the use of wireless sensors that are capable of monitoring the RR continuously. The preliminary version evaluated in this study is a wired one for the sake of sensor’s clinical evaluation.

## 2. Methodology

**Figure 1:**
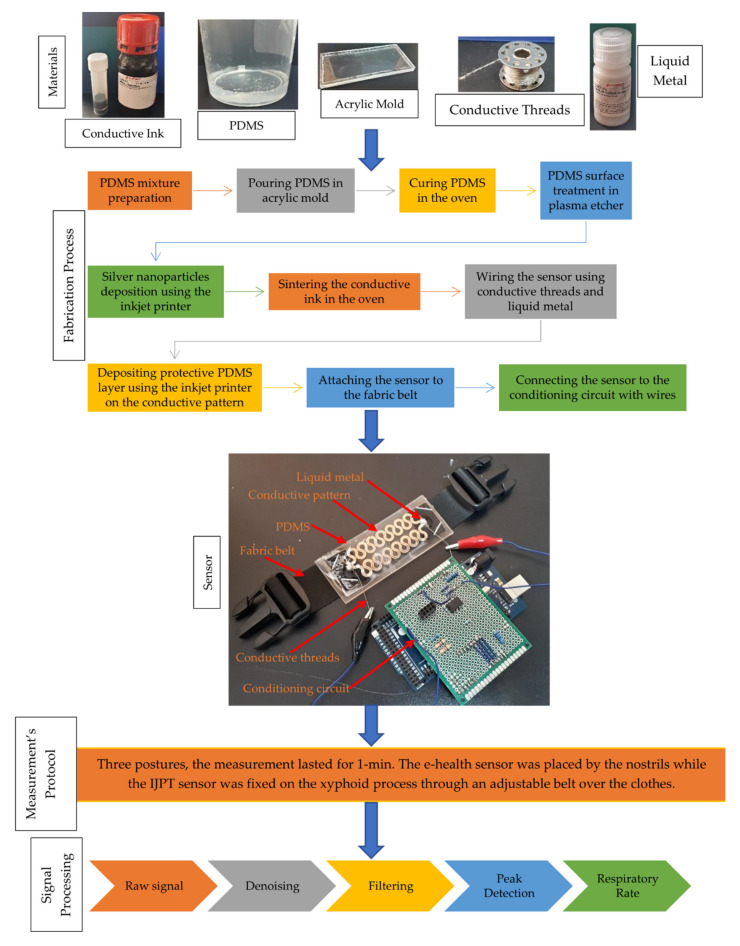
Schematic diagram of the inkjet-printed (IJPT) sensor fabrication process and respiratory rate (RR) extraction on Chronic Obstructive Pulmonary Disease (COPD) patients.

The extraction of the RR using the IJPT on COPD patients consists of several stages, including the fabrication of the IJPT sensor and the signal processing of the RR signal. Figure 1 shows a brief summary of the fabrication process of the IJPT sensor as well as the RR extraction procedure on COPD patients. The following sections elaborate more on these stages.

### 2.1. Ethical Approval

The protocol adhered to the tenets of the Declaration of Helsinki [43]. Each patient had the freedom of choice to participate in this work. The procedure was explained in details with questions answered. Then, the participants signed the consent form, which was reviewed and approved by Jordan University Hospital Ethics Committee (REF 67/2019/6480).

### 2.2. Inkjet-Printed RR Sensor

The fabrication of flexible electronics using inkjet printing technology has gained significant interest in recent years. The RR sensor evaluated in this study was fabricated using this technology where silver nanoparticle ink was deposited on polydimethylsiloxane (PDMS) substrates. The RR was detected by the IJPT sensor via the variations of the resistance of the conductive traces caused by the volumetric change in the ribcage or abdomen areas during the respiration process. In other words, the IJPT sensor acted as a strain gauge sensor. The details of the fabrication process of the IJPT sensor can be found in [16].

### 2.3. Measurements Protocol

Thirty-five patients were included in the study after taking their permissions using the informed consent form. All of them were diagnosed with COPD, and managed with adequate medication and on regular follow-up. Firstly, patients were asked to rest for five minutes while the questionnaire was filled with the health records in patient’s file validated. Then, the respiration rate was measured with the reference e-Health nasal flow sensor and the IJPT sensor simultaneously. The measurement was repeated in 3 different positions: Lying position 45°, sitting upright, and standing. In each position, the measurement lasted for one minute. The e-Health sensor was placed in the nostrils while the IJPT sensor was fixed on the xiphoid process through an adjustable belt over the clothes. It should be noted that the patients were asked not to move while taking the measurements.

The patients included in the study had different GOLD stages of COPD (1, 2, 3 or 4), different age groups (mean ± standard deviation (SD) of age: 55.25 ± 13.76 years) and different smoking status (ex-smoker, quit smoking, or smoker). Some of the limitations faced prevented the measurement of the RR at some postures, such as the inability to take readings from patients on continuous oxygen support as the e-Health sensor was placed by the nostrils. Another limitation was the endurance of some patients to stay in a certain position as it caused fatigue and irritability. In addition, some patients had chest tubes, which caused improper mounting of the IJPT sensor. Despite these limitations, the respiratory signals were successfully recorded in most measurements. A summary of patients’ information, as well as CAGE (Cut-Annoyed-Guilty-Eye) questionnaire summary, can be found in Table A1 and Table A2 in Appendix A, respectively.

### 2.4. Respiration Rate Derivation

**Figure 2:**
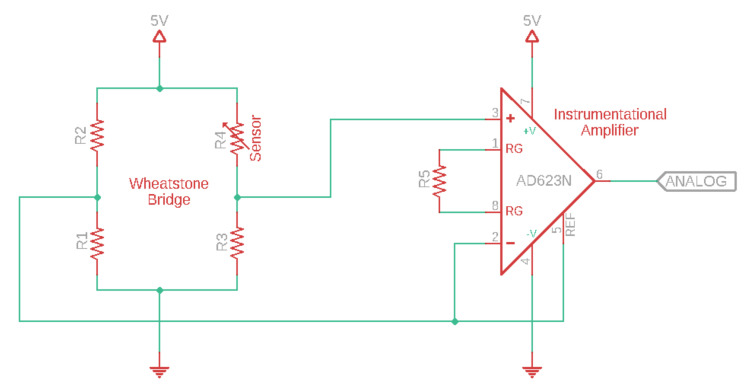
Electrical circuit configuration of the strain gauge sensor.

The strain gauge sensor, whose resistance value varied during respiration, was connected to a Wheatstone Bridge with suitable values for the resistors. The output was connected to an Instrumentational Amplifier with a suitable value for the gain resistor, as shown in Figure 2. The output of the amplifier was connected to an analog input of the Arduino board. The airflow sensor was connected to e-Health Sensor Shield V2.0 for Arduino [44] and connected to the same Arduino board as the strain gauge circuitry. Arduino Mega2560 with an ATMega2560 chip as its microcontroller was used with maximum sampling rate of 15 kSPS at maximum resolution [45,46].

**Figure 3:**
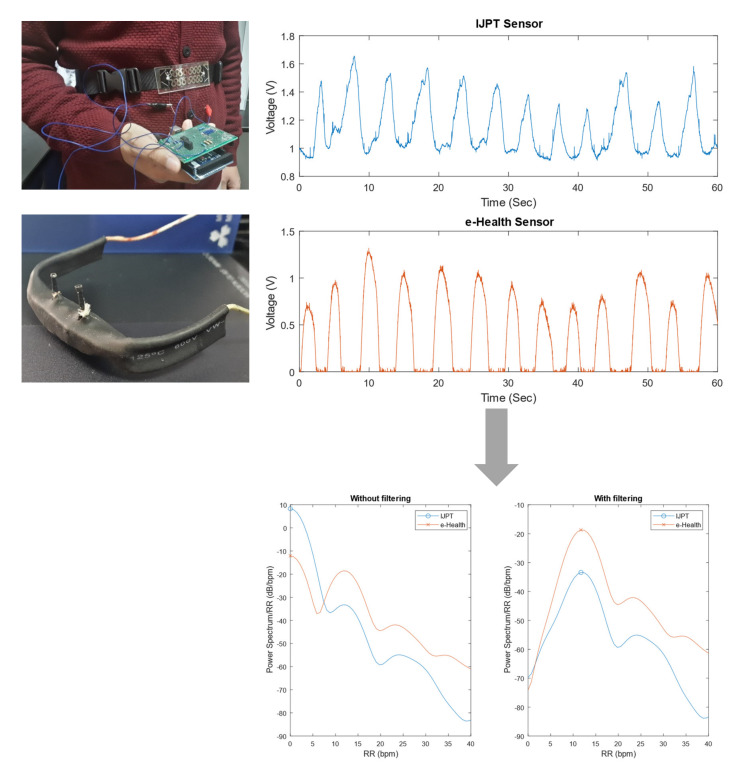
Sample of RR signal processing and RR derivation obtained from patient #9 while sitting using the strain gauge sensor and the e-Health airflow sensor.

The signals of the strain gauge sensor and the airflow sensor were sampled at 100 Hz using the Arduino board, which was connected to a MATLAB/Simulink file to save the data. Afterward, a MATLAB-based bandpass filter was used to filter both signals with cut-off frequencies of 3 and 90 bpm, or 0.05 and 1.5 Hz, respectively, with the function configured to an infinite impulse response. The calculation of the respiratory rate was done by finding the frequency at which the power spectral density of the filtered data had its maximum value. Figure 3 shows the raw RR signals from the IJPT and e-Health sensors as well as power spectral densities of both signals before and after the filtering. The circle and cross indicate the maximum value of the power spectral density of each signal at which the respiratory rate was found.

### 2.5. Statistical Analysis

#### 2.5.1. Data Cleaning

For the data of each measurement, the quality of reference (airflow) respiratory signal was double-checked. If the quality of reference respiratory signal was low (e.g., blurred waveform, missing period longer than 5 s, or missing more than two consecutive respiratory cycles), the data of that measurement were discarded. The considered RR data can be found in Table A3 in Appendix A.

#### 2.5.2. Comparison of RR Values

The RR values derived from inkjet-printed strain gauge (RR_SG_) and airflow (RR_AF_) were compared to investigate if there was any significant difference. Firstly, the Shapiro–Wilk test was performed on RR values to investigate if they followed normal distribution. If both RR_SG_ and RR_AF_ groups followed normal distribution (defined as *p* > 0.05 in Shapiro-Wilk test), the paired T-test was performed to investigate if there was any significant difference (defined as *p* < 0.05) between RR_SG_ and RR_AF_. If normal distribution was not followed in any one group, the Wilcoxon signed-rank test (significant difference defined as *p* < 0.05) was performed as the non-parametric substitute of paired T-test. Considering the missing data in some subjects, the test was performed globally and for each posture, respectively.

#### 2.5.3. Analysis of Errors of RR

The estimation error of RR was calculated as in Equation. (1). The analysis of variance (ANOVA) was performed to investigate if there was any significant effect of individual difference or posture on the results. It has been proven that ANOVA is applicable even if the data deviate from normal distribution [47]. For reliable estimation, the Shapiro–Wilk test was performed to investigate if the error followed normal distribution in three postures respectively. The paired T-test was performed on the data of error derived in different postures if both groups followed normal distribution. Otherwise, the Kruskal–Wallis test was used to investigate if the effect of posture on error was significant (defined as *p* < 0.05), while the Wilcoxon signed-rank test was performed as the non-parametric substitute of paired T-test.

(1) $$E={\mathrm{RR}}_{\mathrm{SG}}-{\mathrm{RR}}_{\mathrm{AF}}$$

#### 2.5.4. Analysis of Relative Errors of RR

The relative error of RR estimation was calculated as in Equation (2). The analysis method is the same as that for error of RR.

(2) $$Er=\frac{{\mathrm{RR}}_{\mathrm{SG}}-{\mathrm{RR}}_{\mathrm{AF}}}{{\mathrm{RR}}_{\mathrm{AF}}}$$

#### 2.5.5. Bland-Altman Analysis

To illustrate the difference between RR_SG_ and RR_AF_ in different postures, the Bland–Altman analysis was performed on RR_SG_ and RR_AF_ for each posture, respectively [48,49]. In signal processing, the resolution of RR was 0.001 bpm. To accurately show and compare the biases, the results of Bland-Altman analysis were rounded to the third significant digit after the decimal point.

#### 2.5.6. Regression Analysis

Linear regression analysis was used to inspect whether the correlation between the RR_SG_ and the RR_AF_ followed a linear correlation or not. The consistency in the RR_SG_ and RR_AF_ was evaluated using the regression coefficient of the linear correlation.

## 3. Results

### 3.1. Comparison of RR Values

The overall distribution of RR_SG_ and RR_AF_ followed normal distribution (*p* = 0.689 and *p* = 0.066 in Shapiro–Wilk test). The paired T-test showed no significant difference between RR_SG_ and RR_AF_ (*p* = 0.572). In sitting posture, normal distribution was followed by RR_AF_ but not RR_SG_ (*p* = 0.104 and *p* = 0.045 in Shapiro–Wilk test, respectively). The Wilcoxon signed-rank test showed no significant difference between RR_SG_ and RR_AF_ (*p* = 0.968). In standing posture, both RR_SG_ and RR_AF_ followed normal distribution (*p* = 0.977 and *p* = 0.652 in Shapiro–Wilk test, respectively). The paired T-test showed no significant difference between RR_SG_ and RR_AF_ (*p* = 0.873). In lying45° posture, both RR_SG_ and RR_AF_ (*p* = 0.629 and *p* = 0.242 in Shapiro–Wilk test, respectively) followed normal distribution. The paired T-test showed no significant difference between RR_SG_ and RR_AF_ (*p* = 0.619).

### 3.2. Analysis of Errors of RR

The ANOVA showed no significant effect of posture (*p* = 0.318) or individual difference (*p* = 0.857) on the measurement error. The normal distribution was followed by the data of standing posture (*p* = 0.516 in Shapiro-Wilk test) but not those of sitting (*p* = 0.007) or lying45° posture (*p* < 0.001). The Wilcoxon signed-rank test showed no significant difference between the data of sitting and standing (*p* = 0.754), sitting and lying45° (*p* = 0.107), or standing and lying45° posture (*p* = 0.944). 

### 3.3. Analysis of Relative Errors of RR

The ANOVA showed no significant effect of posture (*p* = 0.418) or individual difference (*p* = 0.959) on the relative measurement error. The normal distribution was followed by the data of standing posture (*p* = 0.278 in Shapiro-Wilk test) but not those of sitting (*p* = 0.031) or lying45° posture (*p* < 0.001). The Wilcoxon signed-rank test showed no significant difference between the data of sitting and standing (*p* = 0.496), sitting and lying45° (*p* = 0.176), or standing and lying45° posture (*p* = 0.889).

### 3.4. Bland-Altman Analysis

**Figure 4:**
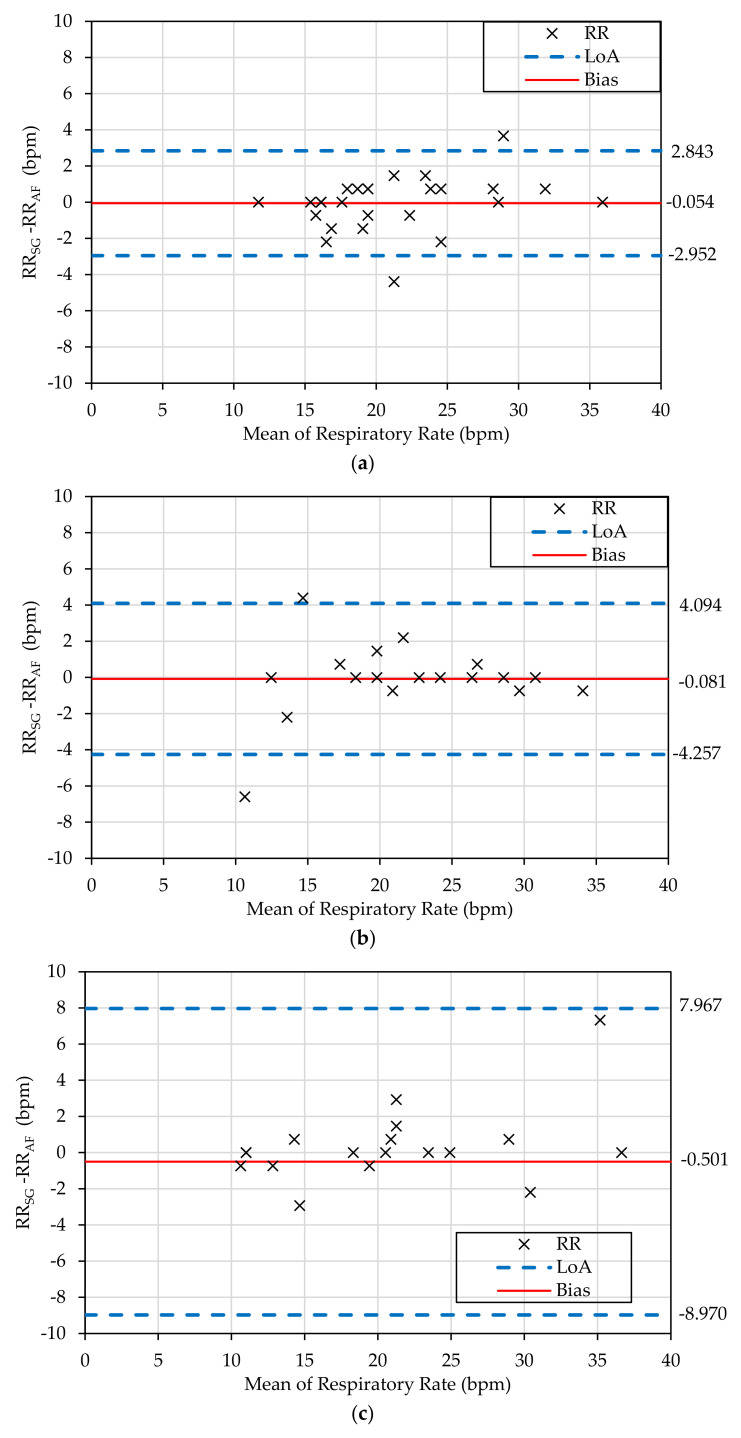
Bland-Altman analysis of the measured respiratory rate of the COPD patients at different postures, namely at (**a**) sitting, (**b**) standing, and (**c**) lying45° posture. Each cross represents the data point of RR measurement. The continuous red line represents the bias, which is the average difference between the RR_AF_ and the RR_SG_ while the dashed blue lines are the limits of agreement where 95% of the data lies in between.

As shown in Figure 4, the smallest bias between the SG-derived and reference RRs was from the measurement in sitting posture (bias: −0.0543 bpm, 95% limits of agreement (LoA): −2.952 to 2.843 bpm). The measurement in standing posture had a similar bias but a wider LoA. The measurement in lying45° posture has the largest bias (−0.501 bpm) and the widest LoA (−8.970 to 7.967 bpm). Thus, the SG measurement was most accurate in sitting posture and least accurate in the lying45° posture.

### 3.5. Regression Analysis

**Figure 5:**
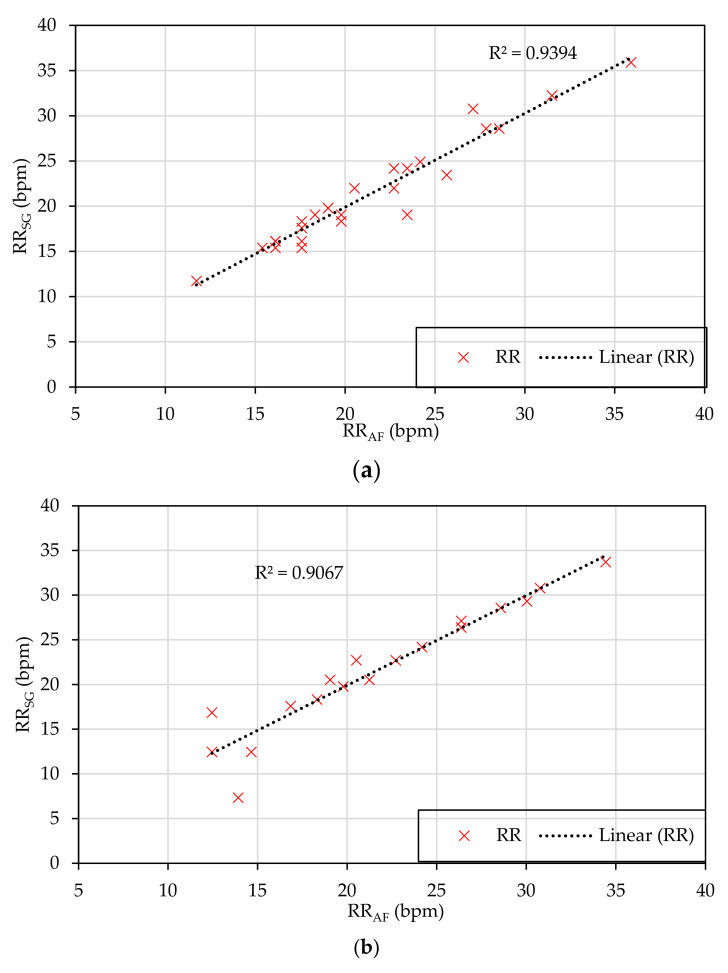

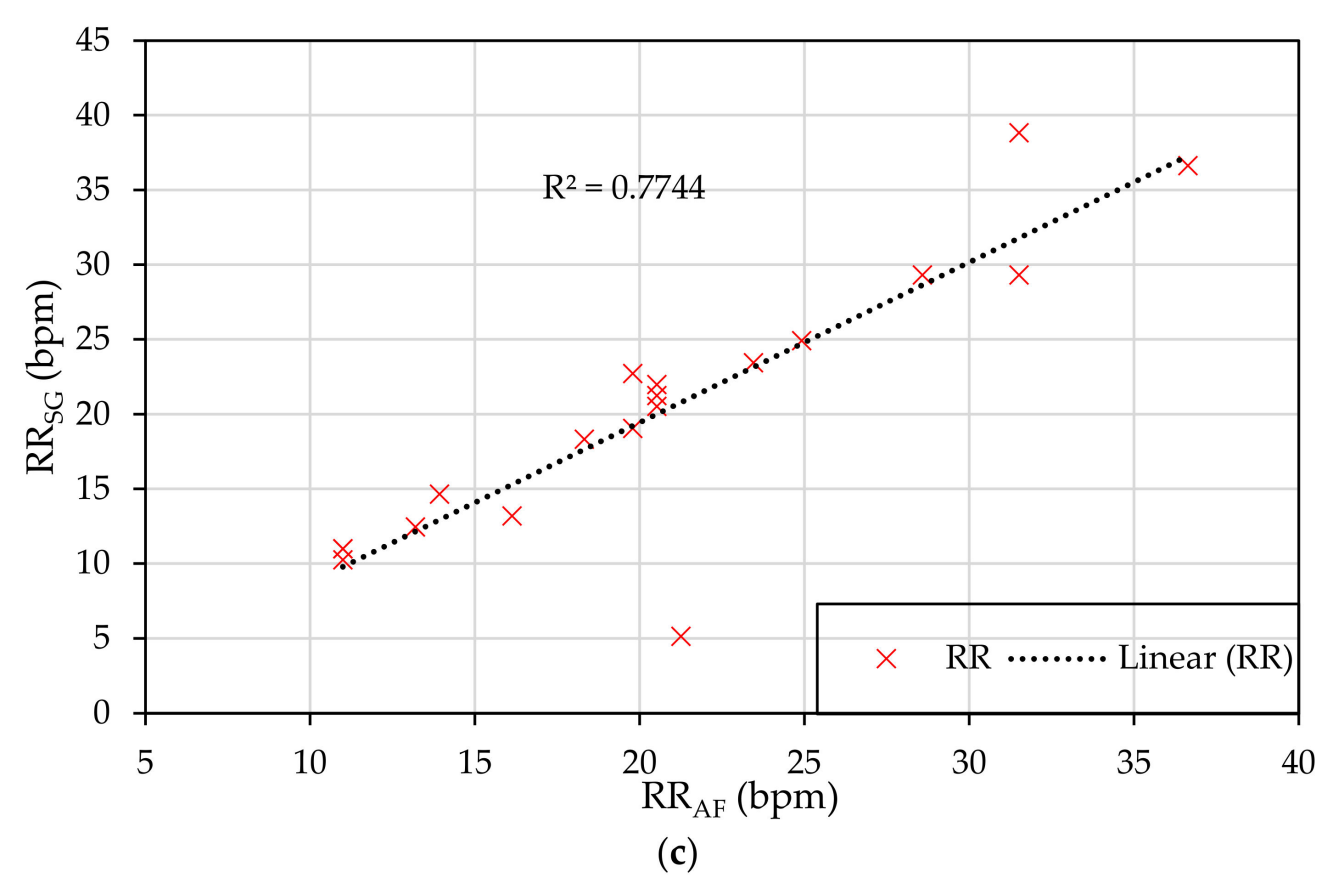
Linear regression analysis of the measured respiratory rate of the COPD patients at different postures namely at: (**a**) Sitting, (**b**) standing, and (**c**) lying45° posture.

The linear regression coefficient between RR_SG_ and RR_AF_ measurements was high for the sitting and standing postures (0.9394 and 0.9067, respectively) while the value was lower in the lying45° posture (0.7744), as shown in Figure 5. The small regression coefficient in the lying45° posture was largely related to the result from an outlier (patient #2) due to losing mounting of the IJPT sensor.

## 4. Discussion

In this work, the IJPT sensor was comprehensively evaluated on 35 COPD patients to measure the RR at different body postures namely standing, sitting at 90°, and lying at 45° positions. The results in this work indicated the high accuracy of the IJPT sensor in RR measurement, with minor differences between different postures.

### 4.1. Measurement of RR on COPD Patients: Difficulties and Approaches

The RR value is one of the most important indicators of several chronic diseases including COPD [50]. RR over 25 bpm is considered as one of the COPD exacerbation signs while the normal range of RR in adults is about 12–20 bpm [40]. RR monitoring of COPD patients is usually carried out in hospitals and clinics via manual counting of breaths, using nasal sensors, or extracted from the electrocardiogram (ECG) signals. The measurement with manual counting is inaccurate [16] while the use of nasal sensors is difficult especially when patients are on continuous oxygen support. Additionally, ECG and nasal sensors are cumbersome and uncomfortable for long-term monitoring. On the other hand, significant progress in the development of WFS has provided the possibility for the convenient, long-term, and low-cost monitoring of vital signs including RR for COPD patients [16,33]. However, there is a lack of validation and clinical evaluation of WFS on COPD patients in different postures, daily activities, and other physiological conditions where the noises could affect the signal quality [31,33,40]. Our results provided a reference for the development of WFS for clinical use.

**Figure 6:**
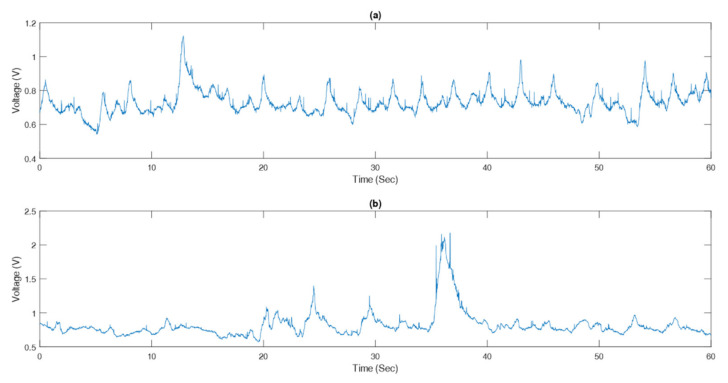
Samples of raw respiratory signals acquired by the IJPT on COPD patient while standing: (**a**) Good signal (test subject #8) and (**b**) deformed signal (test subject #10).

Another challenge is to get accurate estimation of RR in varying breathing patterns. COPD patients usually have an unstable breathing pattern, which causes the variations of RR during measurement. It was observed that RR changed from 15.2 ± 4.3 bpm to 19.1 ± 5.9 bpm during the exacerbation of COPD [24]. COPD patients show characteristic symptoms like breathlessness and cough [51,52]. These symptoms in addition to body posture are associated with the respiratory volume change. Resultantly, the deformed respiratory waveform and amplitude of peaks can lead to the inaccuracy in RR estimation, as observed in this study. Figure 6 shows a comparison between good and deformed respiratory signals measured by the IJPT. It should be noted that the signal quality depends on many factors including the stability of the sensor attachment on the patients. Another factor that affects the signal is the amount of clothing on the patients. Furthermore, the amount of volume change in the abdominal area varies significantly among individuals and also among postures as reported in the literature [17]. These changes in the volume could significantly affect the ability of the IJPT sensor to measure the RR accurately, as seen in the lying45° posture, which is the most challenging posture for RR measurement and yields the results consistent with what was reported in the literature [17]. Furthermore, some COPD patients cannot endure standing or sitting for long periods. Therefore, the comprehensive evaluation of RR monitoring in different circumstances is important for the clinical application of the IJPT sensor [30]. The high measurement accuracy of the IJPT sensor at different postures and among different patients highlighted its potential for reliable monitoring of RR in COPD patients. 

### 4.2. Accuracy of IJPT Sensor: Comparison with Other Sensors

The statistical analysis demonstrated a high accuracy of the IJPT sensor in RR monitoring on COPD patients with good stability at different posture positions. In addition, there was insignificant effect of posture or individual difference on the estimation error. Moreover, the measurement accuracy of the IJPT sensor was comparable with other sensors that have been clinically evaluated on healthy human subjects reported in [35,36,37,38,39]. As shown in Table 1, the IJPT sensor showed higher accuracy in RR monitoring on COPD patients (except in the lying posture, which could be related to losing mounting of the IJPT sensor as aforementioned) than most of the existing sensors.

### 4.3. Applications of IJPT Sensor

Currently, the RR monitoring is often neglected or inaccurately recorded even on patients with respiratory diseases due to the lack of appropriate devices for clinical use [7]. The low fabrication cost, biocompatible substrate, skin-friendly attachment on body surface, and the ease of movement without restrictions in daily activities, as well as the high measurement accuracy of the IJPT sensor make it a promising technology for remote and continuous monitoring of RR for patients with COPD and other respiratory diseases [13]. Reliable RR monitoring for COPD patients using the IJPT sensor could reduce the healthcare cost and the pressure on healthcare facilities especially in low-resource settings such as refugee camps [54]. A recent systematic review disclosed that the majority of physiological monitoring methods for COPD are intermittent with no more than twice a day measurements’ frequency [55]. Acute exacerbations of COPD require intensive care treatment immediately. The evaluation of vital signs is necessary to detect physiological abnormalities (micro events), but patients may deteriorate between measurements [56]. The continuous monitoring of RR on COPD patients is an unmet clinical need that has attracted increasing research focus. Especially, the RR monitoring based on WFS on chest band has been proven with the highest reliability compared with other sensors [57], which makes it possible to provide reliable daily monitoring of COPD based on WFS. Furthermore, the frequency and severity of COPD exacerbation symptoms would be reduced by early detection of abnormal physiological measurements including the respiratory rate [42]. To fight the ongoing pandemic of COVID-19, the combination of internet-of-things (IoT) technology and the IJPT sensor can generate a low-cost, safe, and convenient approach for the remote monitoring, management, and early intervention of the patients where recent studies [58,59] have mentioned that RR could serve as a leading indicator of COVID-19.

### 4.4. Limitations and Future Work

There were some limitations associated with this study. Firstly, the IJPT sensor was tested on 35 COPD patients with only one female and also not all of the measurements at the three postures were available due to the limitations aforementioned in Section 2.3. Therefore, further investigation with more diverse COPD patients is required. Secondly, further clinical evaluation of the IJPT sensor on COPD patients during walking, running, sleeping, and other daily activities can be carried out to expand its application scenarios. Finally, longer time series data should be recorded and clinically evaluated on healthy and non-healthy subjects to further investigate the applicability of the IJPT sensor for continuous monitoring of RR. For future work, an innovative mounting mechanism can be developed as the substitute for the fabric belt to achieve more convenient and reliable attachment in different postures. A wireless platform can be designed to achieve more comfortable measurement for the end user, which will ease the remote and continuous monitoring of RR.

## 5. Conclusions

The results of clinical evaluation on 35 COPD patients in the present work indicated that the IJPT sensor was able to accurately measure the RR at different postures. It can be concluded that the IJPT sensor showed comparable accuracy with other wearable sensors on COPD patients evaluated in the literature with the absolute relative error of 4.49%, 7.29%, and 9.47% at sitting, standing, and lying45° postures, respectively. The IJPT sensor is promising in achieving reliable RR monitoring for COPD patients where the use of this sensor would contribute to mitigating the frequency and severity of COPD exacerbation symptoms by early detection of abnormal physiological measurements including the respiratory rate.

## Figures and Tables

**Table 1:** Comparison between the accuracy and performance of the IJPT sensor and other sensors reported in the literature.

Ref.	Method	Posture	Accuracy Parameter		Protocol	Number of COPD Patients
[33]	Impedance	Activities of daily living protocol	Bias (bpm)	−1.18	Attached to the chest and upper abdomen	44
			LoA (bpm)	−20.07 to 17.72		
	Photoplethysmography (PPG)		Bias (bpm)	3.01	Worn on the wrist with a finger probe	
			LoA (bpm)	−11.17 to 17.19		
	Camera		Bias (bpm)	−3.21	Participant was videoed while in sitting position	
			LoA (bpm)	−12.71 to 6.30		
	Accelerometer		Bias (bpm)	−2.18	Attached to the upper abdomen just below the ribs and taped to the skin	
			LoA (bpm)	−8.63 to 4.27		
	Chest-Band (strain gauge)		Bias (bpm)	−1.60	Chest strap and an electronics module that attaches to the strap	62
			LoA (bpm)	−9.99 to 6.80		
[20]	Capacitive	Rest (lying)	Bias (bpm)	−0.14 bpm	Rest (after exercises)	9
			SD (bpm)	0.28		
[31]	Respiration band (strain gauge)	-	Relative Error (%)	17.43	Attached to the wearable Jacket	30
[53]	Airflow pressure sensor	-	Bias (bpm)	0.046	Hoses attached to the nose	14
			LoA (bpm)	3.865 to 3.957		
This study	Strain gauge	Sitting	Bias (bpm)	−0.0542 bpm	1	35
			LoA (bpm)	−2.951 to 2.842		
			SD (bpm)	1.451		
			Absolute relative error (%)	4.49		
		Standing	Bias (bpm)	−0.0814		
			LoA (bpm)	−4.257 to 4.094		
			SD (bpm)	2.071		
			Absolute relative error (%)	7.29		
		Lying45°	Bias (bpm)	−0.501		
			LoA (bpm)	−8.969 to 6.807.967		
			SD (bpm)	4.227		
			Absolute relative error (%)	9.47		

## Data Availability

The data presented in this study are available in Appendix A.

## Appendix A

**Table A1:** Summary of patients’ information collected.

Patient	Age	Gender	Height (m)	Weight (kg)	Smoking Status	GOLD Diagnosis	First Diagnosed
1	50	M	169	62	Ex-Smoker	3	12/2018
2	51	M	170	71	Smoker	1	11/2001
3	46	M	174	83	Ex-Smoker	3	5/2010
4	30	M	180	62	Smoker	1	N/A
5	79	M	175	98	Ex-Smoker	2	4/2008
6	58	M	162	78	Smoker	3	4/2019
7	39	M	170	85	Quit smoking	2	11/2017
8	56	M	175	90	Smoker	1	8/2013
9	58	M	173	80	Ex-Smoker	2	2/2009
10	26	M	173	84	Ex-Smoker	1	8/2016
11	52	M	176	63	Ex-Smoker	2	2009
12	56	M	170	70	Smoker	N/A	2014
13	47	M	175	80	Ex-smoking	1	7/2018
14	52	M	168	80	Smoker	2	5/2007
15	24	M	173	63	Smoker	1	1/2020
16	42	M	164	85	Smoker	1	10/2014
17	60	M	178	84	Smoker	1	8/2019
18	42	M	165	75	Smoker	2	8/2014
19	49	M	167	64	Smoker	3	1/2020
20	37	M	172	98	Smoker	3	1/2020
21	57	M	167	60	Smoker	3	2/2020
22	70	M	160	50	Smoker	2	2/2020
23	66	M	179	77	Smoker	1	9/2013
24	67	M	175	70	Ex-Smoker	3	5/2001
25	55	M	167	58	Quit smoking	2	11/2019
26	69	M	175	88	Ex-Smoker	2	N/A
27	53	F	167	90	Smoker	1	2/2012
28	58	M	163	74	Ex-Smoker	1	1/2019
29	79	M	174	72	Smoker	3	8/2017
30	67	M	167	62	Smoker	2	8/2017
31	66	M	175	85	Ex-Smoker	1	6/2002
32	67	M	174	69	Smoker	3	6/2014
33	58	M	172	73	Ex-Smoker	3	3/2012
34	75	M	174	68	Smoker	1	11/2009
35	73	M	170	65	Smoker	1	7/2013

N/A: not available. quit smoking: stopped within the last 6 months of interview. Ex-Smoker: stopped smoking after 6 months of interview. A/V fistula: arteriovenous fistula.

**Table A2:** Summary of CAGE (cut-annoyed-guilty-eye) questionnaire.

Patient	Cough	Phlegm (mucus)	Tightness of Chest	Not Able to Climb a Flight of Stairs	Cannot Perform Home Activities	Can Go Out Anytime	Good Sleep	Having Energy
1	frequently	rare	never	always	rare	always	frequently	always
2	rare	rare	sometimes	always	never	always	frequently	rare
3	always	sometimes	sometimes	always	rare	never	sometimes	rare
4	rare	rare	frequently	frequently	never	always	frequently	always
5	always	sometimes	rare	frequently	frequently	rare	sometimes	rare
6	always	always	sometimes	sometimes	sometimes	always	sometimes	never
7	rare	rare	sometimes	rare	rare	rare	frequently	sometimes
8	frequently	sometimes	rare	sometimes	never	always	always	always
9	sometimes	rare	never	never	never	always	frequently	sometimes
10	never	never	never	never	never	always	frequently	always
11	rare	sometimes	never	rare	rare	frequently	frequently	sometimes
12	sometimes	sometimes	never	never	never	frequently	always	always
13	rare	sometimes	never	never	never	always	always	always
14	sometimes	never	never	rare	never	frequently	frequently	frequently
15	rare	never	never	never	never	always	always	always
16	sometimes	frequently	frequently	rare	rare	never	sometimes	frequently
17	rare	rare	rare	never	never	always	frequently	frequently
18	frequently	rare	sometimes	never	never	always	always	frequently
19	frequently	frequently	sometimes	sometimes	rare	frequently	sometimes	frequently
20	frequently	frequently	rare	always	always	sometimes	rare	frequently
21	frequently	rare	always	always	always	always	sometimes	rare
22	always	sometimes	sometimes	sometimes	sometimes	always	frequently	never
23	rare	never	never	never	never	always	frequently	frequently
24	sometimes	never	never	never	frequently	frequently	sometimes	always
25	sometimes	sometimes	frequently	always	always	sometimes	rare	never
26	sometimes	always	sometimes	frequently	frequently	sometimes	sometimes	frequently
27	frequently	rare	never	sometimes	sometimes	always	frequently	frequently
28	always	always	rare	rare	never	always	always	frequently
29	always	frequently	never	always	rare	frequently	rare	frequently
30	always	always	always	always	frequently	rare	rare	never
31	sometimes	never	sometimes	frequently	always	rare	frequently	rare
32	sometimes	never	never	rare	rare	frequently	always	always
33	frequently	always	never	sometimes	frequently	always	rare	sometimes
34	frequently	rare	rare	sometimes	never	always	sometimes	frequently
35	sometimes	rare	sometimes	sometimes	rare	sometimes	frequently	sometimes

**Table A3:** RR_AF_ and RR_SG_ of the COPD measured in addition to the relative error at different body postures.

Patient	Posture	RR_SG_ (bpm)	RR_AF_ (bpm)	Error (bpm)
1	Sitting	28.57	28.57	0
	Standing	30.77	30.77	0
2	Sitting	19.05	18.32	0.73
	Standing	20.51	19.05	1.46
	Lying45°	5.13	21.25	−16.12
3	Sitting	32.23	31.50	0.73
	Standing	29.30	30.04	−0.74
	Lying45°	29.30	31.50	−2.2
4	Sitting	18.32	17.58	0.74
	Standing	7.33	13.92	−6.59
	Lying45°	12.45	13.19	−0.74
5	Sitting	21.98	22.71	−0.73
	Standing	22.71	22.71	0
6	Sitting	19.05	23.44	−4.39
7	Sitting	15.38	17.58	−2.2
	Standing	22.71	20.51	2.2
8	Sitting	30.77	27.11	3.66
	Standing	18.32	18.32	0
9	Sitting	11.72	11.72	0
	Standing	12.45	12.45	0
	Lying45°	10.99	10.99	0
10	Sitting	16.12	17.58	−1.46
	Standing	19.78	19.78	0
11	Sitting	19.05	19.78	−0.73
12	Sitting	18.32	19.78	−1.46
	Standing	24.18	24.18	0
	Lying45°	14.65	13.92	0.73
13	Sitting	16.12	16.12	0
	Lying45°	10.99	10.99	0
14	Sitting	23.44	25.64	−2.2
	Standing	20.51	21.25	−0.74
15	Sitting	21.98	20.51	1.47
	Lying45°	19.05	19.78	−0.73
16	Standing	28.57	28.57	0
	Lying45°	21.25	20.51	0.74
17	Lying45°	13.19	16.12	−2.93
18	Sitting	17.58	17.58	0
19	Sitting	16.12	16.12	0
	Standing	16.85	12.45	4.4
	Lying45°	18.32	18.32	0
20	Sitting	35.90	35.90	0
	Standing	33.70	34.43	−0.73
	Lying45°	36.63	36.63	0
21	Lying45°	22.71	19.78	2.93
22	Sitting	28.57	27.84	0.73
	Lying45°	24.91	24.91	0
23	Sitting	24.91	24.18	0.73
24	Sitting	15.38	16.12	−0.74
	Standing	17.58	16.85	0.73
25	Lying45°	23.44	23.44	0
26	Lying45°	21.98	20.51	1.47
27	Sitting	19.78	19.05	0.73
	Standing	12.45	14.65	−2.2
	Lying45°	10.26	10.99	−0.73
28	Lying45°	38.83	31.50	7.33
29	Sitting	15.38	15.38	0
30	Sitting	24.18	22.71	1.47
31	Lying45°	20.51	20.51	0
32	Sitting	19.78	19.05	0.73
33	Sitting	24.18	23.44	0.74
	Standing	26.37	26.37	0
34	Lying45°	29.30	28.57	0.73
35	Sitting	16.12	16.12	0
	Standing	27.11	26.37	0.74
